# 恩沙替尼治疗EML4-ALK/TP53共突变肺鳞癌1例并文献复习

**DOI:** 10.3779/j.issn.1009-3419.2023.106.03

**Published:** 2023-01-20

**Authors:** Donglai LV, Chunwei XU, Chong WANG, Qiuju SANG

**Affiliations:** ^1^230031 合肥，中国人民解放军联勤保障部队第九〇一医院肿瘤科; ^1^Department of Medical Oncology, 901 Hospital of Joint Logistics Support Force of People Liberation Army, Hefei 230031, China; ^2^210002 南京，南京大学医学院附属金陵医院呼吸与危重症医学科; ^2^Department of Respiratory and Critical Care Medicine, Jinling Hospital, Nanjing University School of Medicine, Nanjing 210002, China

**Keywords:** ALK融合基因, 肺肿瘤, TP53, 恩沙替尼, 洛拉替尼, ALK fusion gene, Lung neoplasms, Tp53, Ensartinib, Lorlatinib

## Abstract

肺鳞癌约占非小细胞肺癌（non-small cell lung cancer, NSCLC）的30%，是第二常见的肺癌组织学类型。具有间变性淋巴瘤激酶（anaplastic lymphoma kinase, ALK）融合突变的NSCLC仅占所有NSCLC病例的2%-5%，且几乎只存在于肺腺癌患者中。因此ALK检测在肺鳞癌患者中不常规进行，个别见于报道的ALK融合突变肺鳞癌患者的靶向治疗效果也不清楚。本例晚期肺鳞癌患者通过二代测序发现存在棘皮动物微管相关蛋白样4（echinoderm microtubule associated protein like 4, EML4）-ALK（V1）和 TP53共突变，2020年12月3日开始口服恩沙替尼，疗效评价为部分缓解（partial response, PR），无进展生存期（progression-free survival, PFS）达19个月，疾病进展后改为洛拉替尼。其中一线恩沙替尼治疗创造了有文献报道以来ALK突变肺鳞癌患者靶向治疗最长的PFS。本文报道了1例接受恩沙替尼治疗的EML4-ALK和TP53共突变肺鳞癌患者，并回顾相关文献，对此类罕见患者的治疗进行了讨论。

## Abstract (en)

Lung squamous cell carcinoma (LSCC) accounts for approximately 30% of non-small cell lung cancer (NSCLC) cases and is the second most common histological type of lung cancer. Anaplastic lymphoma kinase (ALK)-positive NSCLC accounts for only 2%-5% of all NSCLC cases, and is almost exclusively detected in patients with lung adenocarcinoma. Thus, ALK testing is not routinely performed in the LSCC population, and the efficacy of such treatment for ALK-rearranged LSCC remains unknown. Echinoderm microtubule associated protein like 4 (EML4)-ALK (V1) and TP53 co-mutations were identified by next generation sequencing (NGS) in this patient with advanced LSCC. On December 3, 2020, Ensatinib was taken orally and the efficacy was evaluated as partial response (PR). The progression-free survival (PFS) was 19 months. When the disease progressed, the medication was changed to Loratinib. To our knowledge, Enshatinib created the longest PFS of ALK-mutant LSCC patients treated with targeted therapy since literature review. Herein, we described one case treated by Enshatinib involving a patient with both EML4-ALK and TP53 positive LSCC, and the relevant literatures were reviewed for discussing the treatment of this rare disease.

## 1 病例资料

患者为老年女性，73岁，2020年11月8日因“胸背疼痛伴活动后喘憋1月余”就诊于解放军联勤保障部队第九〇一医院。外院胸部X线检查显示双侧肺野内多发块状阴影。2020年11月10日行正电子发射计算机断层扫描（positron emission tomography/computed tomography, PET/CT）显示：（1）右肺上叶尖段软组织肿块，肿块大小为4.7 cm×4.2 cm，^18^F-2-脱氧葡萄糖（^18^fluoro-2-deoxyglucose, FDG）代谢异常增高，最大标准化摄取值（maximum standard uptake value, SUV_max_）为11.9，考虑恶性病变，以右肺上叶周围型肺癌可能性大；（2）双肺多发结节灶，FDG代谢异常增高，肝脏多发略低密度灶，FDG代谢异常增高，双侧肾上腺结节灶，FDG代谢异常增高，双肺门、纵隔内主动脉弓旁、降主动脉旁及右侧腋窝多发肿大淋巴结，FDG代谢异常增高，右上臂肌肉内结节状FDG代谢轻度增高灶，均考虑转移瘤可能性大；（3）右侧胸腔积液；（4）左侧肱骨、双侧锁骨、双侧肩胛骨、胸骨、多根肋骨、多个椎体及附件、骨盆骨及双侧股骨见多个放射性摄取异常增高灶，CT上小部分病灶呈成骨性骨质破坏（图1）。个人史：既往体健，无烟酒史，油烟接触史50年。该病例报道已获得患者家属知情同意。2020年11月8日行肝脏病灶穿刺活检，组织病理学结果示：低分化鳞状细胞癌（图2A）。免疫组化结果：细胞角蛋白7（cytokeratin 7, CK7）（+），P63（+）（图2B、图2C），甲状腺转录因子1（thyroid transcription factor-1, TTF-1）（-）（图2D），P40（+），天冬氨酸蛋白酶A（novel aspartase proteinase A, Napsin A）（-），Ki67（60%+）。程序性死亡配体1（programmed death ligand 1, PD-L1）免疫组织化学染色（22C3）结果：肿瘤比例评分（tumor proportion score, TPS）为0分、综合阳性评分（combined positive score, CPS）为0分。肺癌16个相关基因二代测序（next generation sequencing, NGS）（杭州瑞普基因检测）发现癌细胞存在棘皮动物微管相关蛋白样4（echinoderm microtubule associated protein like 4, EML4）-间变性淋巴瘤激酶（anaplastic lymphoma kinase, ALK）融合（V1亚型，融合功能区域：E13、A20）、 TP53 exon10 p.L348S（c. 1043T>C）突变，丰度为70.04%。结合临床症状及辅助检查结果，该患者诊断为晚期肺鳞癌（cT4N3M1c，IVB期），ALK驱动基因阳性。

**图1:**
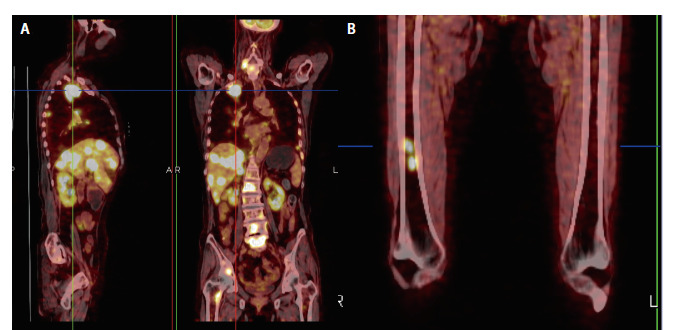
PET/CT扫描结果。A：右肺原发病灶和广泛的转移病灶；B：骨性骨质破坏。

**图2:**
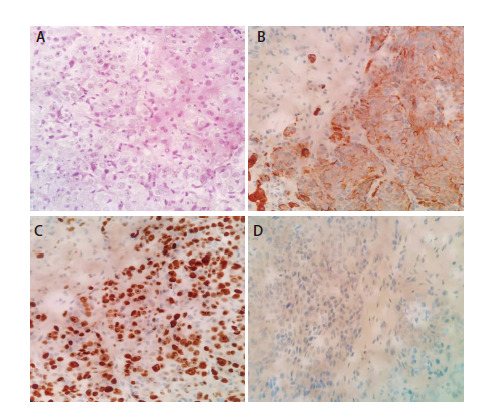
患者肝脏穿刺肿瘤组织标本的组织病理学和免疫组化染色结果。A：苏木精和伊红染色显示低分化鳞状细胞癌（×100）；B、C：CK和P63免疫组化呈强阳性（×100）；D：没有观察到TTF-1的表达（×100）。

完善辅助检查期间患者病情进展迅速，肝功能严重恶化，嗜睡伴进食困难。按照美国东部肿瘤协作组（Eastern Cooperative Oncology Group, ECOG）标准，患者体能状态（performance status, PS）为4分，无法接受常规化疗。糖类抗原（carbohydrate antigen, CA）125和CA199基线水平分别为16,125 U/mL和181.7 U/mL。完善基线影像学评估（图3A）后于2020年12月3日起给予恩沙替尼（225 mg, qd）口服治疗，唑来膦酸注射液治疗骨转移瘤。其中使用恩沙替尼前3日，患者因进入浅昏迷状态用药困难，仅分次服用恩沙替尼200 mg，用药第4日患者神志恢复清醒，恢复正常用药剂量。至2021年2月24日复查CT，显示大多数靶病灶均明显退缩，胸水消退。根据实体瘤疗效评价标准（Response Evaluation Criteria in Solid Tumors, RECIST）1.1评价疗效为部分缓解（partial response, PR）（图3B）。CA125和CA199水平分别降至56 U/mL和8.5 U/mL。患者治疗期间出现2度皮疹，身体状况明显改善，PS评分由4分恢复到1分。用药5.5个月后CT复查显示右肺原发性病灶退缩，弥漫性肝转移病灶消失（图3C）。2022年6月患者出现头痛伴双侧视力下降、肢体进行性无力并发展至瘫痪，遂行头颅磁共振示颅内多发转移癌伴大面积水肿。RECIST 1.1评价疗效为疾病进展（progressive disease, PD）。患者家属拒绝放疗，于2022年7月10日起采用洛拉替尼（100 mg, qd）口服治疗。至7月30日，患者视力、食欲及左侧肢体功能恢复，但出现谵妄、幻时、认知功能障碍等中枢系统不良反应，遂将药物减量（75 mg, qd）。2022年11月19日患者再次出现食欲减退、头痛并伴癫痫发作，患者家属拒绝进一步检查，返回当地维持治疗。

**图3:**
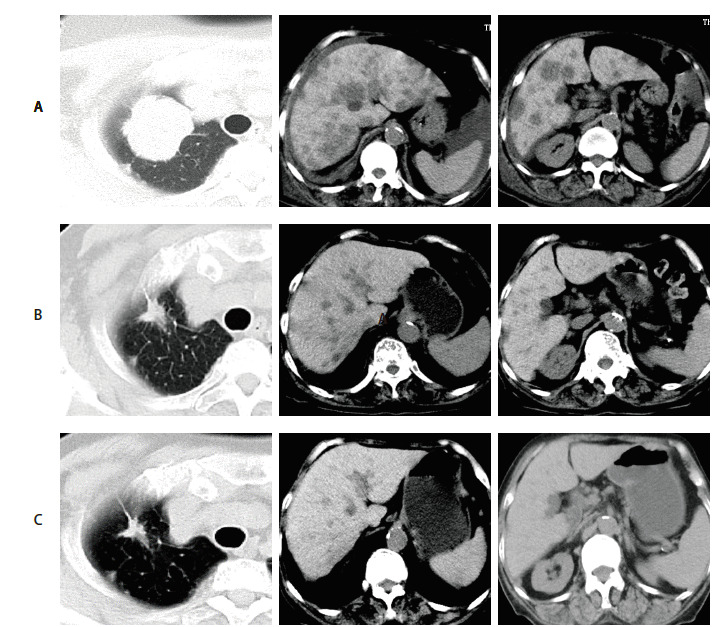
治疗前后胸腹部CT显示的靶病灶变化情况对比。A：恩沙替尼治疗前；B：治疗后83天；C：治疗后169天。

## 2 讨论

在非小细胞肺癌（non-small cell lung cancer, NSCLC）中，ALK融合突变的发生率为2%-5%，且几乎都发生在肺腺癌中^[1]^。先前对亚洲患者的队列研究显示ALK基因融合突变在肺鳞癌中非常罕见，发生率仅为0.7%^[2]^。因此肺鳞状细胞癌中ALK融合突变的靶向治疗也鲜见报道。据我们所知，这是1例报告EML4-ALK融合和TP53共突变的肺鳞癌患者采用第二代ALK抑制剂恩沙替尼治疗的成功案例。

通过检索中国生物医学文献数据库、中文科技期刊数据库、万方数据库以及PubMed、EMBASE等，本文归纳了迄今为止接受靶向药物治疗的ALK融合突变肺鳞癌病例情况^[3⇓⇓⇓⇓⇓⇓⇓⇓⇓-13]^。如表1所示，相关患者只有11例，一线使用的靶向药物包括克唑替尼和阿来替尼。在这些少数病例中，只报道了1例ALK和ROS1双重融合共突变^[11]^，1例罕见包含连接蛋白1的CAP-GLY结构域（CAP-GLY domain containing linker protein 1, CLIP1）-ALK融合突变^[13]^。这两例患者均使用NGS作为较新的技术来取代先前的检测技术，并获得了新的发现。我们认为荧光原位杂交（fluorescence in situ hybridization, FISH）、免疫组织化学（immunohistochemistry, IHC）或实时荧光定量聚合酶链反应（real-time fluorescence polymerase chain reaction, RT-PCR）实验固有的技术局限性导致无法同时检测到更全面的突变靶点，并难以发现罕见的融合突变，NGS检测应被更多地采用。

**表1:** 迄今为止ALK突变肺鳞癌病例靶向治疗情况

First author	Publication year	Age (yr)	Gender	Testing methods	ECOG	Co-mutation	Targeted therapy medicine	Number of treatment lines	Efficacy	PFS (mon)
Srivastava^[3]^	2013	80	Female	FISH	Unclear	Unknown	Crizotinib	1	PD	1.0
Wang^[4]^	2014	55	Female	FISH	Unclear	Unknown	Crizotinib	2	PR	6.3^*^
Mikes^[5]^	2015	36	Male	FISH, PCR	Unclear	Unknown	Crizotinib	2	PR	4.7^*^
Zhang^[7]^	2015	55	Female	IHC	2	Unknown	Crizotinib	2	PR	6.0
Vergne^[8]^	2016	58	Female	IHC, FISH	Unclear	Unknown	Crizotinib	3	PR	7.0
Tamiya^[6]^	2015	78	Male	IHC, FISH	Unclear	Unknown	Alectinib	1	PD	1.4
Wang^[9]^	2016	37	Female	IHC	Unclear	Unknown	Crizotinib	2	PR	9.0^*^
Mamesaya^[12]^	2017	52	Female	IHC, FISH	Unclear	Unknown	Alectinib	2	PR	11.0^*^
Bolzacchini^[10]^	2017	51	Male	Unclear	Unclear	Unknown	Crizotinib/ Ceritinib	2/3	PR/PD	10.0/4.0
Li^[11]^	2017	45	Female	NGS	0	ROS1	Crizotinib	1	PR	3.0
Yuan^[13]^	2022	44	Male	NGS	2	No	Alectinib/ Ensartinib	1/2	SD/PD	4.5/0.5
This case	-	73	Female	NGS	4	TP53	Ensartinib/ Lorlatinib	1/2	PR/SD	19.0/4.5

^*^No progress until the last follow-up. ECOG: Eastern Cooperative Oncology Group; FISH: fluorescence in situ hybridization; IHC: immunohistochemistry; NGS: next generation sequencing; PFS: progression-free survival; PR: partial response; PD: progressive disease; SD: stable diease; RT-PCR: real-time fluorescence polymerase chain reaction.

在ALK基因重排的肺腺癌中，EML4-ALK和TP53共存突变的比例约为5.5%^[14]^，而肺鳞癌患者中没有相关数据。此外，伴随TP53共突变对ALK融合突变NSCLC治疗的影响仍不确定。在表皮生长因子受体（epidermal growth factor receptor, EGFR）突变NSCLC的靶向治疗中，TP53突变将导致靶向药物反应下降、预后变差^[15]^。然而TP53突变与克唑替尼治疗ALK突变肺癌患者疗效之间的关联研究存在矛盾结论^[1,14,16]^。迄今为止，只有一项研究^[1]^报道TP53共突变对ALK突变NSCLC患者的靶向治疗产生负面影响。本例患者经恩沙替尼治疗后，由浅昏迷状态迅速清醒，病灶持续退缩，提示恩沙替尼对于该类型共突变患者有确切的疗效，但仍有必要进一步研究TP53共突变对ALK突变肺鳞癌的长期影响。

另外回顾性分析11例经ALK抑制剂治疗的肺鳞癌患者的一般特征，平均年龄为（53.73±13.74）岁，女性为主（男性4例，女性7例）^[3⇓⇓⇓⇓⇓⇓⇓⇓⇓-13]^。考虑到分子病理学检测手段的时代局限性，2017年以前报道患者的致病基因的共突变无法明确，且靶向药物多采用一代ALK抑制剂克唑替尼为主，治疗线数多为2线。进一步观测疗效可以发现ALK突变的肺鳞癌患者应用克唑替尼或者阿来替尼治疗的PFS均较短，明显差于同类药物在肺腺癌中的药物反应时间。此外这些患者中的老年人对药物的反应和预后更差。在队列病例中^[3,6]^ 70岁以上的2例患者接受了克唑替尼或阿来替尼的一线治疗，但疾病均快速进展，只维持了1个月和1.4个月的PFS。然而本例患者73岁，一般状况差，且EML4-ALK融合突变为疗效相对较差的V1亚型，结果采用恩沙替尼一线治疗不但取得PR的良好疗效，且PFS达到19个月，远超此前报道的所有同类病例，表明恩沙替尼或许是此类患者一线治疗的较好选择。后期患者出现颅内进展，未行基因检测情况下予以洛拉替尼治疗，见效，虽维持时间较短，但也为该类型患者的二线治疗选择提供了部分依据。

本文描述1例EML4-ALK（V1）融合和TP53高丰度共突变的老年肺鳞癌患者，使用恩沙替尼治疗迅速见效，并创造了有文献报道以来ALK阳性肺鳞癌患者靶向治疗最长的PFS。本文的研究结果可能对该类型肺癌患者的治疗提供一些启示。
